# Safety and efficacy of minimally invasive gastrectomy for older patients with gastric cancer after neoadjuvant chemotherapy and immunotherapy: a propensity score-matched analysis

**DOI:** 10.1186/s12877-024-05193-w

**Published:** 2024-07-15

**Authors:** Hao Cui, Zhen Yuan, Wenquan Liang, Bo Cao, Lin Chen, Jianxin Cui, Bo Wei

**Affiliations:** 1 School of Medicine, Nankai University, Tianjin, 300071 China; 2 Department of General Surgery & Institute of General Surgery, Chinese PLA General Hospital First Medical Center, Beijing, 100853 China

**Keywords:** Gastric cancer, Older patients, Minimally invasive surgery, Neoadjuvant immunotherapy, Complications

## Abstract

**Background:**

The effect of neoadjuvant immunotherapy on minimally invasive gastrectomy (MIG) in older patients with gastric cancer remains controversial. This study aimed to evaluate the safety, and efficacy of MIG for older patients who underwent neoadjuvant chemotherapy and immunotherapy (NICT).

**Methods:**

The clinical data of 726 older patients aged over 65 years who underwent upfront MIG or MIG after NICT in the Department of General Surgery, Chinese PLA General Hospital First Medical Center between Jan 2020 and Nov 2023 were retrospectively analyzed. Propensity score-matched (PSM) analysis at a ratio of 1:2 was performed to reduce bias from confounding patient-related variables, short- and long-term outcomes were compared between the two groups.

**Results:**

The baseline characteristics were comparable between 61 patients in the NICT-MIG group and 114 patients in the MIG group after PSM (*P* > 0.05). The major pathological response (MPR) rate and pathological complete response (pCR) rate were 44.2% and 21.3%, respectively, in the NICT-MIG group. Patients in the NICT-MIG group had longer operation times (*P* = 0.005) and postoperative days (*P* = 0.030) than those in the MIG group. No significant differences were found in intraoperative bleeding, number of retrieved lymph nodes, first flatus day, R0 resection rate, overall postoperative complication (POC) morbidity, severe POC morbidity, 2-year overall, and recurrence-free survival between the MIG and NICT-MIG groups (*P* > 0.05). Multivariate logistic analysis revealed that an estimated blood loss > 200 mL (*P* = 0.010) and a lymphocyte-to-monocyte ratio (LMR) ≤ 3.25 (*P* = 0.006) were independent risk factors for POCs after MIG in older patients.

**Conclusion:**

The safety, and efficacy of NICT-MIG were comparable to those of upfront MIG in older patients with GC. Patients with an estimated blood loss > 200 mL or an LMR ≤ 3.25 should be carefully evaluated for an increased risk of POCs in older patients who undergo MIG.

**Trial registration:**

Chinese Clinical Trial Registry (Registration Number: ChiCTR2400086827).

**Supplementary Information:**

The online version contains supplementary material available at 10.1186/s12877-024-05193-w.

## Background

Gastric cancer (GC) is one of the most common malignant tumors and is the second most common cause of both cancer incidence and cancer-related death in China [1]. As the phenomenon of aging intensifies, the proportion of older patients with GC is gradually increasing [2]. Due to poor physical status and comorbidities, it is essential to conduct comprehensive geriatric assessments and select optimal perioperative treatments for older patients [3].

In recent years, minimally invasive gastrectomy (MIG), represented by laparoscopic and robotic gastrectomy, has been widely applied to cure GC due to its several advantages, including less surgical incision, less blood loss and comparable survival benefits compared with open gastrectomy (OG) [4, 5]. The advantages of MIG have attracted surgeons to focus on its safety and efficacy in older patients. Hikage M et al. conducted a propensity score-matched analysis and demonstrated that patients who underwent MIG achieved comparable short-term outcomes and better overall and relapse-free survival than patients who underwent OG [6]. Li’s study also showed that MIG was a feasible and safe procedure for older patients, in contrast to MIG for nonolder patients and OG for older patients [7]. Moreover, our previous study revealed that MIG with intracorporeal anastomosis for older patients was safe and improved postoperative quality of life [8].

Neoadjuvant therapy before surgery is emerging as a highly regarded treatment for patients with advanced GC. The combination of neoadjuvant chemotherapy and immunotherapy (NICT) is one of the most mainstream therapies, resulting in better tumor regression, increasing the proportion of patients who underwent R0 resection, and providing potential survival benefits [9, 10]. However, NICT might result in treatment-related adverse events (TRAEs), decreasing immune and nutritional status, especially in older patients. Moreover, the edema and fibrillation of peri-gastric tissues caused by NICT also increase surgical difficulty [11]. Although the current study retrospectively demonstrated the safety and feasibility of MIG after NICT [12], it is still uncertain whether MIG can be safely conducted after NICT for older patients. This study aimed to evaluate the therapeutic effects of NICT, safety and efficacy of MIG after NICT compared with those of upfront MIG and to determine the risk factors that affect postoperative complications in older patients who undergo MIG.

## Methods

### Patients

The inclusion criteria were as follows: (1) aged over 65 years, (2) histologically demonstrated gastric adenocarcinoma based on preoperative gastroscopy, (3) accepted laparoscopic or robotic gastrectomy plus D2 lymphadenectomy, (4) pathological tumor stage of p/ypTNM 0-III based on the International Union Against Cancer/American Joint Committee on Cancer guidelines (8th edition), (5) treatment with simultaneous neoadjuvant chemotherapy and immunotherapy in the NICT-MIG group, (6) Eastern Cooperative Oncology Group (ECOG) score 0–1, and (7) available integrated clinical and pathologic data. Patients with an American Society of Anesthesiologists (ASA) grade > III, a history of remnant gastric cancer or gastrointestinal stromal tumor, combined other organ resection, or who received other preoperative regimens, such as radiotherapy and targeted therapy, were excluded from the study.

**Fig. 1:**
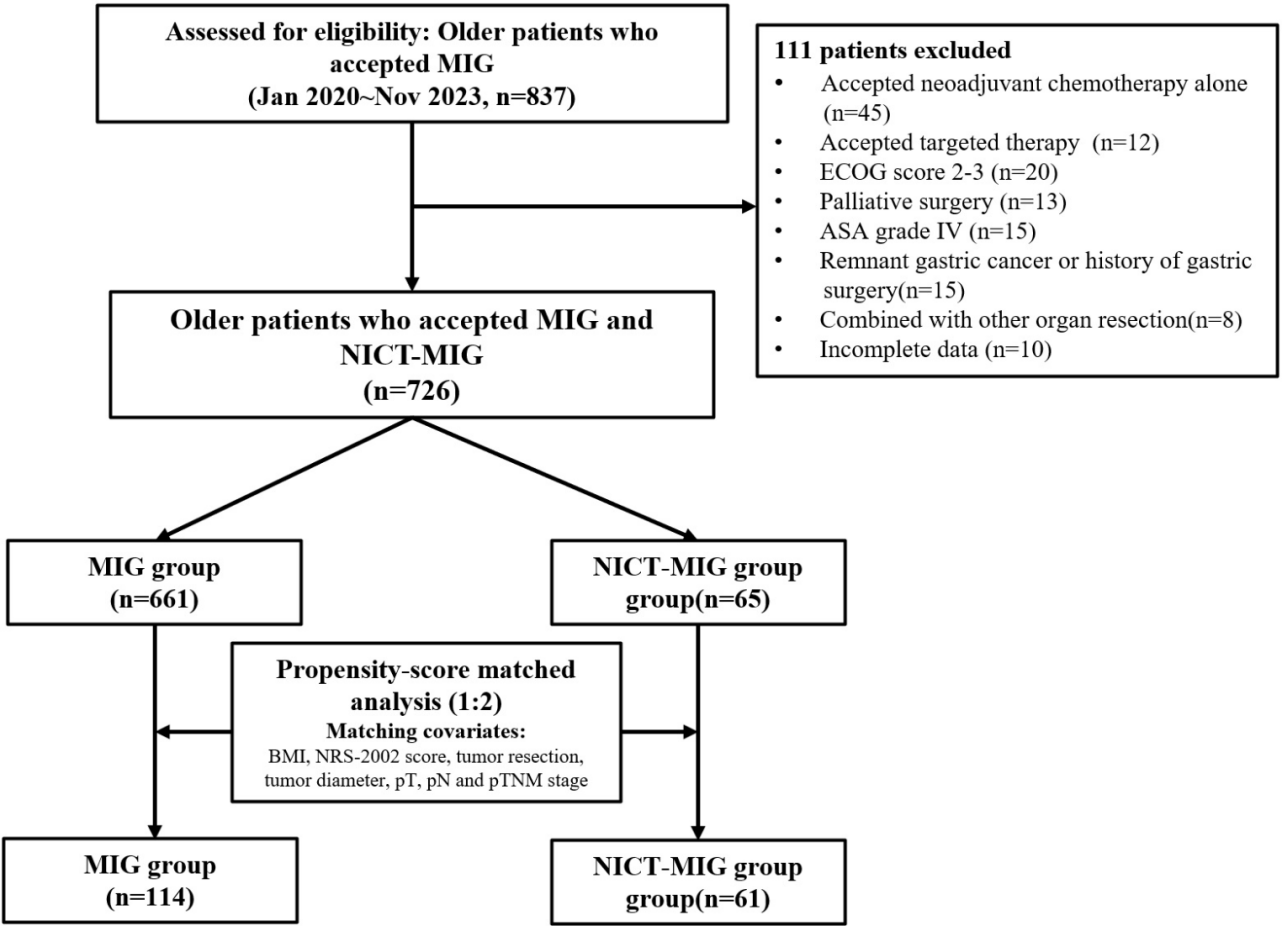
Flow chart of this study

According to the criteria mentioned above, we retrospectively collected clinical and pathological data from 726 older patients with GC (65 patients in the NICT-MIG group and 661 patients in the MIG group) from Jan 2020 to Nov 2023 at the Chinese PLA General Hospital First Medical Center. The flow chart is shown in Fig. 1.

### Propensity score matching (PSM) analysis

**Fig. 2:**
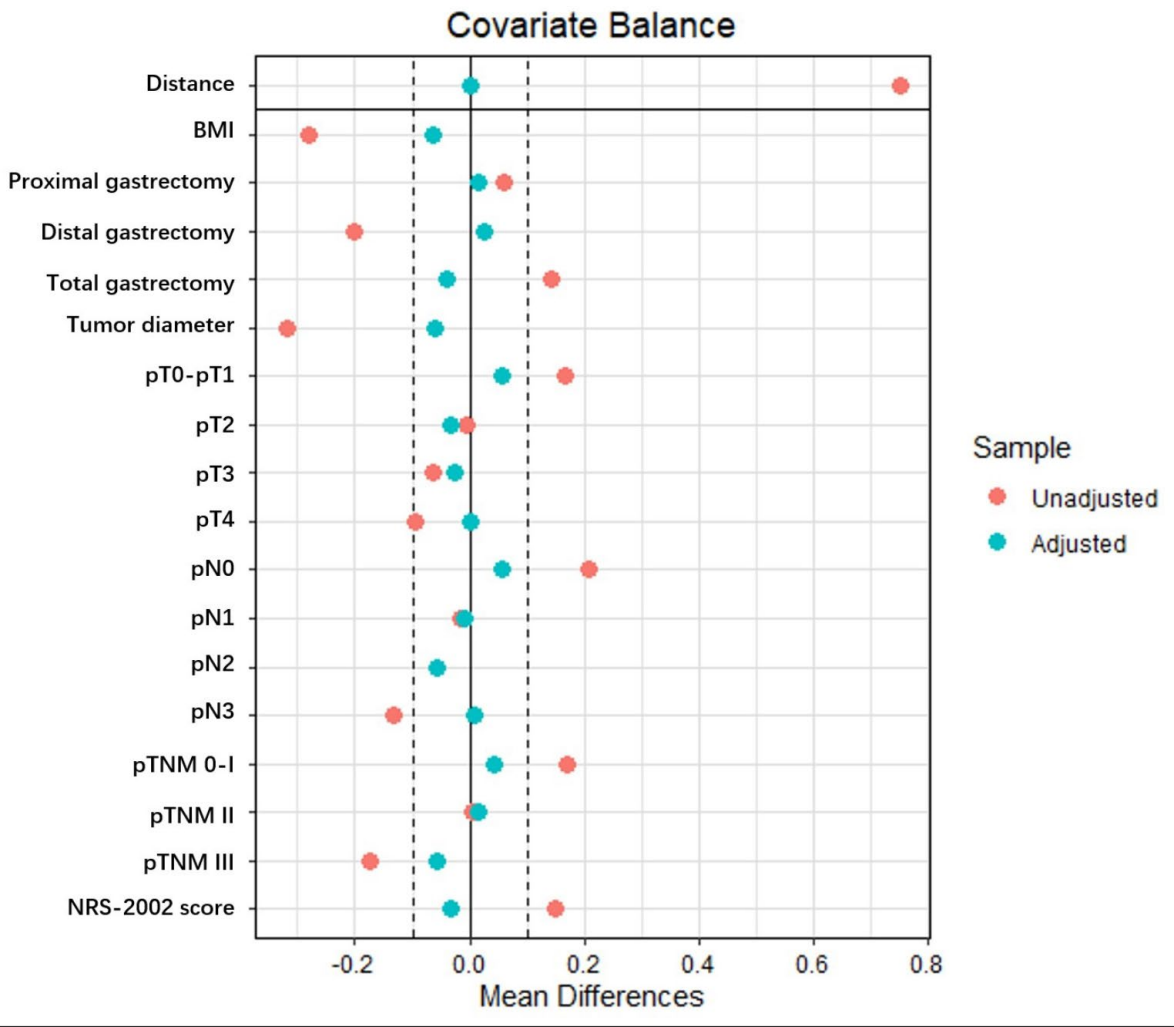
Covariate balance before and after PSM

**Fig. 3:**
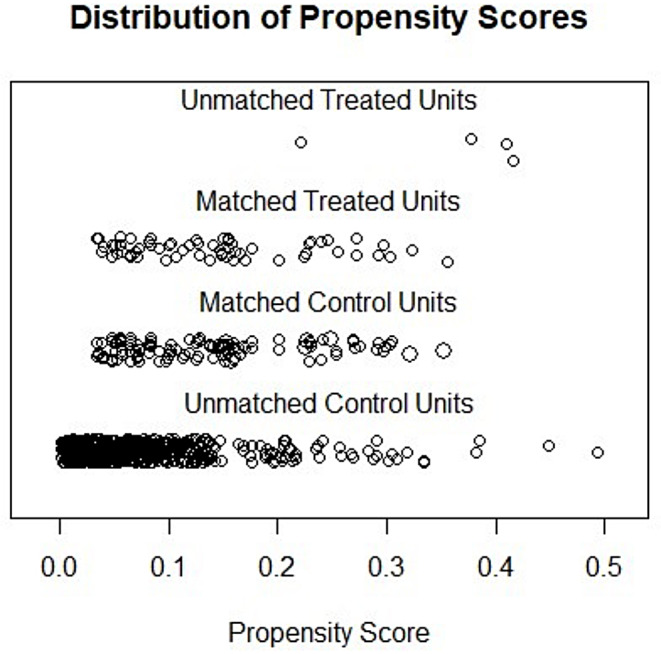
Distribution of propensity scores before and after PSM

In this study, we employed PSM analysis using R statistical software (version 4.2.2; R Foundation, Vienna, Austria) to minimize baseline significant differences. The propensity score for each patient was determined by a logistic regression model based on clinical indices, including body mass index (BMI), nutritional risk screening-2002 (NRS-2002) score, tumor resection, tumor diameter, and p/ypT, p/ypN and p/ypTNM stages. Patients in the NICT-MIG and MIG groups were matched at a 1:2 ratio using the nearest neighbor matching approach with an optimal caliper width of 0.20 without replacement. The covariate balance and distribution of propensity scores before and after PSM are shown in Figs. 2 and 3. After matching, 61 patients in the NICT-MIG group and 114 patients in the MIG group were included in the final analyses.

### Treatment with NICT and evaluation of the therapeutic effect

The regimens of neoadjuvant chemotherapy included SOX (S-1 at 60 mg twice daily from Day 1 to 14 combined with oxaliplatin at 130 mg/m^2^ on Day 1, with 21 days being a cycle), XELOX (capecitabine at 1,000 mg/m^2^ twice daily from Day 1 to 14 combined with oxaliplatin at 130 mg/m^2^ on Day 1, with 21 days being a cycle), AS (S-1 at 60 mg twice daily from Day 1 to 14 combined with nab-paclitaxel at 125 mg/m^2^ on Day 1 and Day 8), capecitabine (1,000 mg/m^2^ twice daily from Day 1 to 14, with 21 days being a cycle) or S-1 (60 mg twice daily from Day 1 to 14, with 21 days being a cycle). The regimens used for neoadjuvant immunotherapy in this study were all PD-1 inhibitors, including nivolumab (360 mg), tislelizumab (200 mg), camrelizumab (200 mg) and sintilimab (200 mg), which were administered intravenously every 21 days as a cycle.

The therapeutic effect was evaluated every 2 cycles by a multidisciplinary team using enhanced abdominal CT according to the Response Evaluation Criteria in Solid Tumors (version 1.1) [13]. The severity of the treatment-related adverse events (TRAEs) was estimated by the Common Terminology Criteria for Adverse Events (CTCAE - Version 5.0) [14]. We used Becker’s standard to define the tumor regression grade (TRG) from pathological results, which included the following: (1) TRG1a, no residual tumor cells; (2) TRG1b, < 10% residual tumor cells; (3) TRG2, 10–50% residual tumor cells; and (3) TRG3, > 50% residual tumor cells [15]. Pathologic complete response (pCR) and major pathological response (MPR) were defined as TRG1a and TRG1a/1b, respectively.

### Surgical approach

MIG was performed within 4–6 weeks after the last cycle in the NICT-MIG group, while patients in the MIG group underwent surgery without any neoadjuvant therapies. Laparoscopic or robotic radical gastrectomy plus D2 lymphadenectomy was conducted based on the surgeons’ individual selection. The standardized surgical procedures were in accordance with the Japanese Gastric Cancer Treatment Guidelines (version 5) [16], the Chinese Guidelines for Laparoscopic Gastrectomy (2023 Edition) [17], and the Chinese Expert Consensus on Robotic Gastrectomy (2015 Edition) [18]. All surgeons had abundant experience with MIGs and overcome the learning curve.

After the intracorporeal procedure, a longitudinal incision less than 10 cm in the upper abdomen was used to remove the specimen, and then intracorporeal or extracorporeal anastomosis was performed according to the surgeons’ discretion. The brief modalities of anastomosis were as follows: (1) Billroth II combined with Braun or Roux-en-Y anastomosis for distal gastrectomy; (2) esophageal–gastric anastomosis, tubular anastomosis, or double-flap anastomosis for proximal gastrectomy; and (3) Roux-en-Y anastomosis for total gastrectomy.

### Perioperative indices

We collected several preoperative laboratory indices that might predict postoperative complications, including the following: (1) platelet/lymphocyte ratio (PLR): platelet count/lymphocyte count, (2) lymphocyte/monocyte ratio (LMR): lymphocyte/monocyte ratio, (3) Onodera’s prognostic nutritional index (PNI) score: 10 × albumin (g/dL) + 0.005 × lymphocyte count/mm^3^, and (4) Albumin (ALB) level.

To evaluate surgical safety and postoperative recovery, estimated blood loss, operation time, R0 resection, first flatus days, postoperative hospitalization days, postoperative overall complications and severe complication rates were recorded. The Clavien–Dindo classification was used to demonstrate the severity of postoperative complications (POCs) (overall POCs: POCs with a Clavien–Dindo classification ≥ Grade II; severe POCs: POCs with a Clavien–Dindo classification ≥ Grade IIIa) [19].

### Follow-up

The follow-up data of all patients were regularly recorded by outpatient service or telephone. For the first three years, follow-up was conducted every six months, and then every year thereafter. The missed follow-up rate was 5.1% (9/175) until May 24th, 2024. The median follow-up periods were 25.0 months (95% CI: 20.19–29.81 months) in the MIG group and 24.0 months (95% CI: 21.06–26.94 months) in the NICT-MIG group.

### Statistical analysis

Statistical analyses were conducted using R software, version 4.2.2 (R Foundation for Statistical Computing) and SPSS (version 26.0; SPSS, Chicago, IL, USA). For continuous variables following a normal distribution, the mean ± standard deviation.

was used for representation, and *Student’s t* test was performed to compare the differences. For continuous data with a skewed distribution, the median (interquartile range) [M(IQR)] was utilized, and statistical analysis was carried out using the *Mann‒Whitney U* test. Categorical data are expressed as frequencies (percentages), and the chi-square test or Fisher’s exact test was used to evaluate differences between two groups. The log-rank test was used to compare long-term survival, and curves were drawn using the Kaplan–Meier method. Binary logistic regression and Cox analyses were conducted, and factors with *P* < 0.1 in the univariate analysis were included in the multivariate analysis. A significance level of *P* < 0.05 was considered statistically significant.

## Results

### Baseline characteristics before and after PSM

Table 1 presents the patient characteristics of the entire cohort (*n* = 726) and propensity score-matched cohort (*n* = 175). After comparing the two groups before PSM, we found that BMI (*P* = 0.035), NRS-2002 score less than 3 (*P* = 0.031), extent of tumor resection (*P* = 0.008), p/ypT stage (*P* = 0.007), p/ypN stage (*P* = 0.009), p/ypTNM stage (*P* = 0.009), and tumor diameter (*P* = 0.035) were significantly different between the NICT-MIG and MIG groups.

After PSM, no significant differences were found in sex, age, BMI, NRS-2002 score, age-adjusted Charlson comorbidity index (aCCI) score, ASA grade, history of abdominal surgery, surgical approach, extent of tumor resection, p/ypT stage, p/ypN stage, p/ypTNM stage, or tumor diameter between the two groups (*P* > 0.05). The pathological characteristics were also comparable, with no significant difference after PSM, as shown in Table 2.

**Table 1:** Baseline characteristics between NICT-MIG and MIG group before and after PSM

Baseline characteristics	Before PSM			After PSM		
	MIG group(*n* = 661)	NICT-MIG group(*n* = 65)	*P* Value	MIG group(*n* = 114)	NICT-MIG group(*n* = 61)	*P* Value
**Sex [n (%)]**			0.787			0.502
Male	524(79.3)	53(81.5)		87(76.3)	50(82.0)	
Female	137(20.7)	12(18.5)		27(23.7)	11(18.0)	
**Age (year**,** mean ± SD)**	69.3 ± 2.79	69.1 ± 2.90	0.587	69.6 ± 2.94	69.3 ± 2.84	0.499
**BMI(kg/m**^**2**^,**mean ± SD)**	23.1 ± 3.11	24.0 ± 3.26	0.035	23.4 ± 3.32	23.2 ± 3.06	0.654
**NRS-2002 score [n (%)]**			0.031			0.911
<3	301(45.5)	20(30.8)		35(30.7)	20(32.8)	
≥ 3	360(54.5)	45(69.2)		79(69.3)	41(67.2)	
**aCCI score [n (%)]**			0.218			0.467
≤ 5	499(75.5)	44(67.7)		84(73.7)	41(67.2)	
>5	162(24.5)	21(32.3)		30(26.3)	20(32.8)	
**History of abdominal surgery [n (%)]**			0.849			0.951
No	558(84.4)	56(86.2)		99(86.8)	52(85.2)	
Yes	103(15.6)	9(13.8)		15(13.2)	9(14.8)	
**ASA Grade [n (%)]**			0.706			0.762
I	6(0.9)	0(0)		1(0.9)	0(0)	
II	554(83.8)	54(83.1)		94(82.5)	51(83.6)	
III	101(15.3)	11(16.9)		19(16.7)	10(16.4)	
**Surgical approach [n (%)]**			0.353			0.207
Laparoscopic	590(89.3)	55(84.6)		104(91.2)	51(83.6)	
Robotic	71(10.7)	10(15.4)		10(8.8)	10(19.4)	
**Tumor resection [n (%)]**			0.008			0.955
Proximal	133(20.1)	17(26.2)		28(24.6)	16(26.2)	
Distal	316(47.8)	18(27.7)		33(28.9)	18(29.5)	
Total	212(32.1)	30(46.2)		53(46.5)	27(44.3)	
**pT [n (%)]**			0.007			0.814
T0-T1	165(25.0)	27(41.5)		38(33.3)	24(39.3)	
T2	84(12.7)	8(12.3)		18(15.8)	7(11.5)	
T3	338(51.1)	29(44.6)		56(49.1)	29(47.5)	
T4	74(11.2)	1(1.5)		2(1.8)	1(1.6)	
**pN [n (%)]**			0.009			0.818
N0	259(39.2)	39(60.0)		61(53.5)	36(59.0)	
N1	103(15.6)	9(13.8)		16(14.0)	8(13.1)	
N2	119(18.0)	8(12.3)		21(18.4)	8(13.1)	
N3	180(27.2)	9(13.8)		16(14.0)	9(14.8)	
**pTNM [n (%)]**			0.009			0.814
0-I	204(30.9)	31(47.7)		48(42.1)	28(45.9)	
II	170(25.7)	17(26.2)		29(25.4)	16(26.2)	
III	287(43.4)	17(26.2)		37(32.5)	17(27.9)	
**Tumor diameter[cm**,** M(IQR)]**	4.0(2.5–5.5)	3.0(2.0–5.0)	0.035	3.0(2.0-5.5)	3.0(2.0–5.0)	0.885

*Abbreviation* NICT: Neoadjuvant immunotherapy plus chemotherapy; MIG: Minimally invasive gastrectomy; ASA: American Association of Anesthesiologists; BMI: Body mass index; aCCI: Age-adjusted Charlson Comorbidity Index; NRS-2002: Nutritional risk screening-2002; PSM: Propensity-Score Matched

**Table 2:** Pathological characteristics of MIG group and NICT-MIG group after PSM for older patients

Pathological characteristics	MIG group(*n* = 114)	NICT-MIG group(*n* = 61)	*P* Value
**Differentiation [n (%)]**			0.079
Well /Moderate	77(67.5)	33(54.1)	
Poor/Undifferentiated	37(32.5)	28(45.9)	
**Singal-ring adenocarcinoma [n (%)]**			0.096
No	87(76.3)	53(86.9)	
Yes	27(23.7)	8(13.1)	
**Nerve invasion [n (%)]**			0.965
No	80(70.2)	43(70.5)	
Yes	34(29.8)	18(29.5)	
**Vascular invasion [n (%)]**			0.271
No	83(72.8)	49(80.3)	
Yes	31(27.2)	12(19.7)	
**MMR status [n (%)]**			0.276
pMMR	109(95.6)	55(90.2)	
dMMR	5(4.4)	6(9.8)	

*Abbreviation* NICT: Neoadjuvant immunotherapy plus chemotherapy; MIG: Minimally invasive gastrectomy; MMR: Mismatch repair

### Therapeutic effects and TRAEs in NICT-MIG

Table 3 shows the preoperative treatment and tumor response in the NICT-MIG group. Most patients (*n* = 45, 73.8%) accepted ≤ 4 treatment cycles. A total of 39 (63.9%), 16 (26.2%), 3 (4.9%), and 3 (4.9%) patients received SOX, XELOX, AS and other chemotherapy regimens, respectively. According to the RECIST 1.1 criteria, 5 patients (8.2%) achieved CR, 33 patients (54.1%) achieved PR, 19 patients (31.1%) achieved SD, and 4 patients(6.6%) had PD. The objective response rate (ORR) and disease control rate (DCR) were 62.3% and 93.4%, respectively. Based on Becker’s standard, 13 (21.3%) patients achieved pCR with TRG1a, and 27 (44.2%) patients achieved MPR with TRG1a/1b.

As mentioned in Table 4, the three most common TRAEs affecting therapeutic safety were nausea and vomiting (26.2%), thrombocytopenia (24.6%), and leukopenia (23.0%). The overall rates of TRAEs and severe TRAEs were 83.6% and 27.9%, respectively. No treatment-related deaths occurred in the NICT-MIG group.

**Table 3:** Preoperative treatment and tumor response in the NICT-MIG group

Treatment characteristics	NICT-MIG group(*n* = 61)
**Treatment cycle [** ***n (%)]***	
≤ 4	45(73.8)
>4	16(26.2)
**Chemotherapy regimen [n (%)]**	
SOX	39(63.9)
XELOX	16(26.2)
AS	3(4.9)
Others	3(4.9)
**Radiological response [n (%)]**	
CR	5(8.2)
PR	33(54.1)
SD	19(31.1)
PD	4(6.6)
**ORR [n (%)]**	38(62.3)
**DCR [n (%)]**	57(93.4)
**TRG [n (%)]**	
1a	13(21.3)
1b	14(23.0)
2	23(37.7)
3	11(18.0)
**pCR rate [n (%)]**	13(21.3)
**MPR rate [n (%)]**	27(44.2)

*Abbreviation* NICT: Neoadjuvant immunotherapy plus chemotherapy; MIG: Minimally invasive gastrectomy; CR: Complete response; PR: Partial response; SD: Stable disease; PD: Progressive disease; ORR: Objective response rate; DCR: Disease control rate; TRG: Tumor regression grade

**Table 4:** Treatment-related adverse events of NICT-MIG group for older patients

Treat-related adverse events (TRAEs)	NICT-MIG group(*n* = 61)		
	Total	Grade1-2	Grade3-4
Leukopenia	14	10	4
Thrombocytopenia	15	11	4
Neutropenia	10	9	1
Nausea and vomiting	16	12	4
Dysphagia	1	1	0
Anorexia	6	6	0
Anemia	3	2	1
Aminotransferase increased	5	3	2
Cholangitis	1	1	0
Alopecia	1	1	0
Medicamentosa	1	1	0
Neurotoxicity	1	0	1
Peripheral neuropathy	2	2	0
Hypothyroidism	2	2	0
Diarrhea	2	2	0
**Overall TRAEs rate [n (%)]**	51(83.6)		
**Severe TRAEs rate [n (%)]**	17(27.9)		

*Abbreviation* NICT: Neoadjuvant immunotherapy plus chemotherapy; MIG: Minimally invasive gastrectomy; TRAEs: Treatment-related adverse events

### Surgical safety and postoperative recovery

As shown in Table 5, compared with the MIG group, the NICT-MIG group had longer operation times (235.20 ± 53.89 min vs. 210.55 ± 55.07 min, *P* = 0.005) , more volume of postoperative day 3 drainage [200(100-400)ml vs. 100(75-150)ml, P=0.019], and postoperative hospitalization days [9.0 (7.0–10.0) days vs. 7.0 (6.75-9.0) days, *P* = 0.030]. No significant differences were found in estimated blood loss [100.0 (50.0-100.0) ml vs. 100.0 (50.0-100.0) ml, *P* = 0.568], number of retrieved lymph nodes [26.5 (18.75–36.25) vs. 25.0 (20.5–40.0), *P* = 0.650], first flatus days [3.0 (3.0–4.0) d vs. 4.0 (3.0–4.0), *P* = 0.724], or the R0 resection rate (100% vs. 95.1%, *P* = 0.076) between the MIG and NICT-MIG groups. For patients who needed adjuvant therapy, the rate of completion of the total cycle was significantly greater in the NICT-MIG group than in the MIG group (90.1% vs. 72.7%, *P* = 0.012).

**Table 5:** Comparison of surgical characteristics and postoperative recovery between MIG and NICT-MIG groups

Variable	MIG group(*n* = 114)	NICT-MIG group(*n* = 61)	*P* Value
**Operation time**,** min(mean ± SD)**	210.55 ± 55.07	235.20 ± 53.89	0.005
**Estimated blood loss**,** mL(median**,** IQR)**	100.0(50.0-100.0)	100.0(50.0-100.0)	0.568
**Retrieved lymph nodes**,** n (median**,** IQR)**	26.5(18.75–36.25)	25.0(20.5–40.0)	0.650
**Fist flatus day**,** d (median**,** IQR)**	3.0(3.0–4.0)	4.0(3.0–4.0)	0.724
**Radical resection [n (%)]**			0.076
R0	114(100.0)	58(95.1)	
R1	0(0)	3(4.9)	
**Postoperative day 3 drainage volume**,** mL (median**,** IQR)**	100(75–150)	200(100–400)	0.019
**Gastrointestinal reconstruction [n (%)]**			0.361
Esophageal-gastric anastomosis	22(19.3)	11(18.0)	
Tubular anastomosis	3(2.6)	5(8.2)	
Double-flap anastomosis	3(2.6)	0(0)	
Billroth II + Braun	17(14.9)	12(19.7)	
Roux-en-Y for distal gastrectomy	16(14.0)	6(9.8)	
Roux-en-Y for total gastrectomy	53(46.5)	27(44.3)	
**Postoperative hospitalization day**,** d (median**,** IQR)**	7.0(6.75-9.0)	9.0(7.0–10.0)	0.030
**Total complication rate [n (%)]**	20(17.5)	15(24.6)	0.267
**Clavien-Dindo Classification**			
**Grade II**			
Anastomosis hemorrhage	2	0	
Atrial fibrillation	1	2	
Pancreatic fistula	0	1	
Premature ventricular beats	0	1	
Thrombocytopenia	0	1	
Anastomosis leakage	2	1	
Gastroparesis	1	0	
Intestinal obstruction	2	0	
Aminotransferase increased	0	1	
Anemia	5	1	
Acute cerebral infarction	1	0	
Pneumonia	3	0	
Hypoproteinemia	2	6	
**Grade III**			
Anastomosis leakage	1	0	
**Grade V**			
Acute pulmonary embolism	0	1	
Heart failure	1	0	
**Severe complication rate [n (%)]**	2(1.8)	1(1.6)	1.000
**Completion rate of perioperative treatment cycles [n (%)]**	48/66(72.7)	55/61(90.1)	0.012

*Abbreviation* NICT: Neoadjuvant immunotherapy plus chemotherapy; MIG: Minimally invasive gastrectomy

### Postoperative complications and risk factors

There was no significant difference between the MIG cohort and the NICT-MIG cohort regarding the overall complication rate (17.5% vs. 24.6%, *P* = 0.267) or severe complication rate (1.8% vs. 1.6%, *P* = 1.000) within 30 days after surgery. The most common POCs were anemia and hypoproteinemia in the MIG and NICT-MIG groups, respectively. One patient died due to heart failure in the MIG group, while another patient died as a result of acute pulmonary embolism in the NICT-MIG group.

Table 6 shows the albumin(ALB) changes in the serum levels before and after surgery. Patients in the NICT-MIG group were more prone to have lower serum ALB levels than were those in the MIG group at postoperative Day 3, even though no significant difference was found in the preoperative serum ALB levels between the two groups (Supplementary Fig. 1). A higher incidence of hypoproteinemia with Clavien-Dindo classification II in older patients in the NICT-MIG group was also reported (9.8% vs. 1.8%, *P* = 0.039).

**Table 6:** Changes of albumin level between NICT-MIG and MIG group

	MIG group(*n* = 114)	NICT-MIG group(*n* = 61)	*P* value
Albumin level before surgery, g/L (mean ± SD)	39.68 ± 4.02	39.15 ± 3.69	0.394
Albumin level at postoperative day 3, g/L (mean ± SD)	33.98 ± 3.23	32.65 ± 3.20	0.010
Hypoproteinemia	2(1.8)	6(9.8)	0.039

*Abbreviation* NICT: Neoadjuvant immunotherapy plus chemotherapy; MIG: Minimally invasive gastrectomy; SD: Standard deviation

Table 7 presents the results of logistic regression, which aimed to explore the risk factors for POCs in older patients with GC receiving MIG. Univariate analysis revealed that non-R0 resection [OR (95% CI): 8.424 (0.741–95.716), *P* = 0.086], estimated blood loss [OR (95% CI): 4.313 (1.527–12.184), *P* = 0.006], LMR [OR (95% CI): 3.692 (1.705–7.998), *P* = 0.001], , and PLR [OR (95% CI):.

1.965 (0.922–4.189), *P* = 0.080] were associated with POCs (*P* < 0.1). NICT before MIG was not an independent risk factor [OR (95% CI): 1.533 (0.719–3.266), *P* = 0.269] for POCs. Multivariate logistic analysis revealed that an estimated blood loss > 200 mL [OR (95% CI): 4.232(1.409–12.708), *P* = 0.010] and a lymphocyte-to-monocyte ratio (LMR) ≤ 3.25 [OR (95% CI): 3.132(1.397–7.026), *P* = 0.006] were found to be independent risk factors for POCs after MIG in older patients.

**Table 7:** Uni- and multivariate logistic analysis for overall postoperative complications after MIG in older patients

Factor	Univariate analysis		*P* value	Multivariate analysis		*P* value
	OR	95%CI		OR	95%CI	
Sex			0.855			
Male	1.000					
Female	1.086	0.448–2.636				
Age (years)			0.650			
<70	1.000					
≥ 70	1.187	0.566–2.491				
BMI (kg/m^2^)			0.618			
<25	1.000					
≥ 25	0.808	0.349–1.871				
NICT			0.269			
No	1.000					
Yes	1.533	0.719–3.266				
aCCI score			0.676			
≤ 6	1.000					
>6	1.187	0.531–2.651				
NRS-2002 score			0.684			
<3	1.000					
≥ 3	0.850	0.388–1.863				
Surgical approach			0.554			
Laparoscopic	1.000					
Robotic	1.389	0.468–4.122				
R0 resection			0.086			0.096
Yes	1.000			1.000		
No	8.424	0.741–95.716		8.994	0.679-119.048	
Tumor resection			0.524			
Proximal	1.000					
Distal	1.046	0.402–2.724	0.926			
Total	0.660	0.262–1.659	0.377			
Tumor diameter(cm)			0.840			
<3	1.000					
≥ 3	0.840	0.399–1.770				
Operation time(min)			0.632			
<240	1.000					
≥ 240	1.208	0.559–2.611				
Estimated Blood loss(mL)			0.006			0.010
≤ 200	1.000			1.000		
>200	4.313	1.527–12.184		4.232	1.409-12.708	
Albumin level(g/L)			0.429			
<35	1.000					
≥ 35	1.675	0.466–6.015				
PNI score			0.813			
>45	1.000					
≤ 45	1.097	0.590–2.367				
LMR			0.001			0.006
>3.25	1.000			1.000		
≤ 3.25	3.692	1.705–7.998		3.132	1.397–7.026	
PLR			0.080			0.186
<148	1.000			1.000		
≥ 148	1.965	0.922–4.189		1.748	0.766–3.940	

*Abbreviation* NICT: Neoadjuvant immunotherapy plus chemotherapy; MIG: Minimally invasive gastrectomy; BMI: Body mass index; aCCI: Age-adjusted Charlson Comorbidity Index; NRS-2002: Nutritional risk screening-2002; PLR: Platelet-lymphocyte ratio; LMR : Lymphocyte-monocyte ratio; PNI: Onodera’s prognostic nutritional index

### Long-term survival

**Fig. 4:**
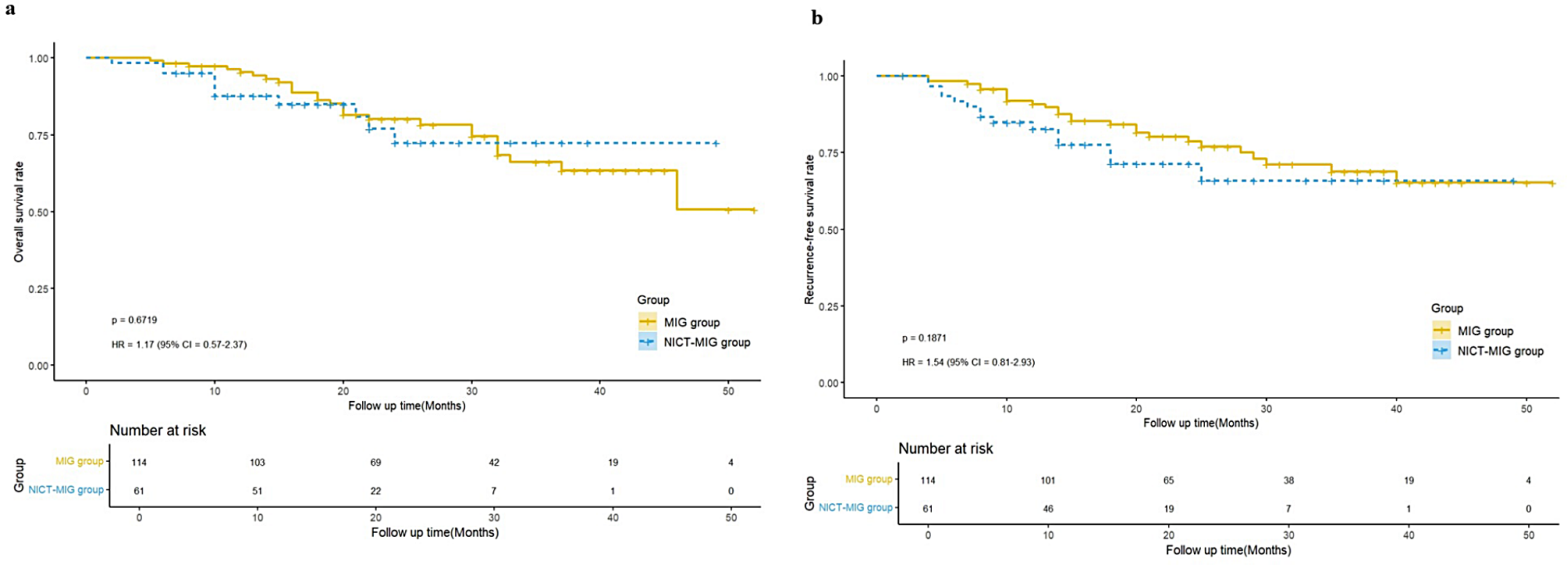
Kaplan-Meier curve of overall survival and recurrence-free survival between NICT-MIG and MIG group a: Kaplan-Meier curve of overall survival between two groups; b. Kaplan-Meier curve of recurrence-free survival between two groups

Figure 4 shows the Kaplan‒Meier curves of overall survival (OS) and recurrence-free survival (RFS) between the two groups. We found that the 2-year OS and 2-year RFS rates were both comparable in the MIG and NICT-MIG groups (80.0% vs. 72.4%, log-rank χ^2^ = 0.179, *P* = 0.672; 78.7% vs. 71.3%, log-rank χ^2^ = 1.741, *P* = 0.187). Subgroup analysis revealed that patients with ypTNM stage III in the NICT-MIG group had poorer RFS than patients in the MIG group (*P* = 0.043) (Supplementary Fig. 2). Cox analysis revealed that LMR ≤ 3.25 and poor p/ypTNM stage were independent risk factors for OS in older patients who underwent MIG (*P* = 0.015, *P* = 0.001). Non-R0 resection and poor p/ypTNM stage were found to be independent risk factors for RFS in older patients who underwent MIG (*P* = 0.004, *P* < 0.001) (Supplementary Tables 1 and 2).

## Discussion

This retrospective PSM study mainly focused on the short- and long-term outcomes of older patients who underwent upfront MIG or MIG after NICT, aiming to explore the safety and efficacy of NICT and MIG after NICT for older patients with GC.

NICT has played a pivotal role in treating locally advanced GC in recent years, offering advantages such as a greater probability of tumor regression, downstaging and potential long-term survival benefits. However, older patients typically exhibit a weaker immune system, leading to a reduction in the number of T cells and decreased levels of tumor-infiltrating lymphocytes. This may impact the clinical benefits that older patients derive from NICT. Nishijima TF’s study demonstrated similar survival benefits between younger and older patients who received immune checkpoint inhibitors (ICIs) [20]. Wu Q et al. reported that cancer patients older than 75 years failed to achieve remarkable benefits from ICIs in terms of both overall survival and progression-free survival [21]. Our study revealed that 13 (21.3%) and 27 (44.2%) older patients with GC in the NICT-MIG group achieved pCR and MPR, respectively. These findings indicate a comparable tumor response in older patients, aligning with previous studies conducted across all age populations [22]. We also found the comparable 2-year OS and RFS between two groups, especially for pTNM satege I patients undergoing upfront MIG and ypTNM stage 0-I patients who had better tumor response in the NICT-MIG group. Thus, our results suggest that NICT induces better tumor regression in older patients with GC, potentially leading to improved survival, which was similar with the older patients who underwent upfront MIG with the same pathological TNM stage.

The TRAEs resulting from NICT may compromise the physical condition and impact perioperative safety, particularly in older patients. A study by Manji GA reported that 57.1% of patients experienced severe (grade 3 or higher) TRAEs during NICT, and 3 older patients (over 75 years old) experienced grade V TRAEs [23]. A meta-analysis demonstrated that the incidence rates of total TRAEs and grade 3 to 4 TRAEs were 89.1% and 34.4%, respectively [24]. In our study, the overall rates of TRAEs and severe TRAEs in the NICT-MIG group were 83.6% and 27.9%, respectively, which are similar to findings from previous studies. These results indicate the medical safety considerations associated with NICT for older patients with GC.

Operation time and estimated blood loss are crucial indicators reflecting surgical safety and predicting POCs. We observed that patients in the NICT-MIG group had a longer operation time than did those in the upfront MIG group (235.20 ± 53.89 min vs. 210.55 ± 55.07 min, *P* = 0.005). Although no significant difference was found in the estimated blood loss between the two groups (*P* = 0.568), multivariate logistic analysis revealed that an estimated blood loss of > 200 mL [OR (95% CI): 4.232(1.409–12.708), *P* = 0.010] was an independent risk factor for POCs in older patients who underwent MIG. These results indicate that the adverse effects of NICT, such as increased tissue fragility, severe exudative edema, myelosuppression, and immune system weakening, increase surgical difficulty and pose a potential risk of POCs.

Moreover, the longer postoperative hospitalization days in the NICT-MIG group than in the MIG group also indicate delayed postoperative recovery attributable to NICT. According to our clinical experience and observation, for older patients who undergo NICT-MIG, prolonged operation time (*P* = 0.005) has a greater impact on cardiopulmonary function, and postoperative recovery may be appropriately extended to ensure the perioperative safety. Moreover, due to obvious exudate and edema in the surgical area after NICT, the postoperative drainage volume (postoperative Day 3) in the NICT-MIG group was significantly greater than that in the direct surgery group (*P* = 0.019), making it easier for patients to develop related complications such as hypoproteinemia (9.8% vs. 1.8%, *P* = 0.039), and the time for tube removal was extended, leading to a relatively longer postoperative hospital stay. Therefore, it is imperative for surgeons to conduct comprehensive perioperative evaluations and perform meticulous surgical procedures to ensure the safe application of NICT-MIG in older patients.

The application of MIG in older patients has been widely acknowledged, as evidenced by current studies [25, 26]. However, it is still controversial whether it is equally reliable to conduct MIG after NICT. A meta-analysis revealed that surgical complications were more common with neoadjuvant therapy for GC [27]. A multicenter study demonstrated that the incidence of POCs and the proportion of perioperative textbook outcomes were comparable between patients who underwent laparoscopic gastrectomy after NICT and those who received neoadjuvant chemotherapy alone- [28]. In this study, no significant differences were found between the upfront MIG and NICT-MIG cohorts regarding overall and severe POC rates, indicating the safety and feasibility of NICT-MIG for older patients. Moreover, we also need to be vigilant regarding MIG/NICT-MIG-related deaths, including acute pulmonary embolism and heart failure, which are prone to occur in older patients [29].

The LMR is considered a promising biomarker for predicting therapeutic effects and long-term prognosis in patients with GC. Most studies have demonstrated that a lower LMR might be associated with poor prognosis in GC patients [30, 31]. Shigeo Tokumaru et al. also reported that the LMR could predict the tumor response of GC patients who received nivolumab [32]. With respect to predicting postoperative complications, Selçuk Gülmez et al. demonstrated that the preoperative LMR had the best ability to predict postoperative infections after gastrectomy [33]. Hsu JT’s study showed that GC patients with lower LMR had higher surgical mortality rates [34]. In our study, we selected an LMR of 3.25 as the cutoff value according to the ROC curve. Multivariate analysis revealed that a preoperative LMR ≤ 3.25 was an independent risk factor for both POCs and overall survival in older patients with GC who underwent MIG. This interesting finding led us to hypothesize that the decrease in lymphocyte counts reflects the impairment of physical immunity, while elevated monocyte levels often imply increasing levels of tumor-associated macrophages (TAMs), which synergistically affect postoperative recovery, increase the risk of complications, and cause poor survival, especially in older patients with comorbidities or malnutrition [35].The correlation between nutritional status and long-term prognosis in older patients has been widely studied. The serum ALB level and Onodera’s PNI score are representative indices that can reflect the nutritional status of older patients. In this study, we found that the serum ALB level on postoperative Day 3 was significantly lower in the NICT-MIG group than in the MIG group, even though no significant difference was found in the preoperative serum ALB level between the two groups. A decrease in nutritional status caused by NICT or MIG might affect adjuvant therapy, and increase the likelihood of disease progression and deterioration of immunocompetence. Kanda M et al. demonstrated that the prognostic significance of the PNI was more apparent in patients with stage II GC and in those who received adjuvant chemotherapy [36]. We found that a lower preoperative PNI (PNI ≤ 45) was not significantly associated with more POCs, poor OS, or RFS by univariate analysis for older patients who underwent MIG. We attributed this interesting phenomenon to two aspects: (1) NICT has resulted in a better tumor response and improved the completion rate of total cycles of perioperative treatment in older patients, which may offset the adverse effects of adjuvant chemotherapy caused by poor nutritional status after surgery; and (2) all enrolled patients in this study underwent nutritional assessment and timely supplementation of enteral or parenteral nutrition in accordance with the European Society for Clinical Nutrition and Metabolism (ESPEN) guidelines [37], which weakened the impact of nutritional status on prognosis.

This study has several inherent limitations. First, some potential bias may still exist even though we used PSM analysis to balance the baseline characteristics between the two groups. Second, we lack in-depth analysis of some key positive results due to the limited length and understanding. Based on future large-scale research, we will conduct more in-depth exploration of interesting phenomena found in this article. Third, because of the limited sample size in this retrospective study, we did not standardize the regimen or number of cycles of NICT. Further large-scale prospective studies should be conducted to eliminate the potential effect of different types of NICT and explore the longer prognosis for older patients, providing high-level evidence for the broader application of NICT in older patients with GC.

## Conclusion

To the best of our knowledge, this is the first study dedicated to assessing the safety and efficacy of NICT-MIG in older patients with GC. Our findings indicate that older patients who undergo NICT can achieve equally good tumor responses, such as ORR, pCR and MPR, with an acceptable incidence of TRAEs. Despite a longer operation time and longer postoperative hospitalization days in the NICT-MIG group, no significant differences were observed in terms of the R0 resection rate, estimated blood loss, number of retrieved lymph nodes, first flatus day, overall POCs, severe POCs, 2-year OS or RFS compared with those in the upfront MIG group. These findings underscore the safety and efficacy of NICT-MIG for older patients. For patients with estimated blood loss > 200 mL and an LMR ≤ 3.25, it is recommended that surgeons prioritize perioperative management to mitigate the risk of POCs.

### Electronic supplementary material

Below is the link to the electronic supplementary material.

Supplementary Material 1

Supplementary Material 2

Supplementary Material 3

## Data Availability

All datasets generated for this study are included in the article and available from corresponding authors upon reasonable request.

## Abbreviations

NICT: Neoadjuvant immunotherapy combined with chemotherapy
GC: Gastric cancer
LAGC: Locally advanced gastric cancer
MIG: Minimally invasive gastrectomy
PSM analysis: Propensity-score matched analysis
ASA: American Association of Anesthesiologists
BMI: Body mass index; aCCI: Age-adjusted Charlson Comorbidity Index
NRS-2002: Nutritional risk screening-2002
pCR: Pathological complete response
MMR: mismatch repair
MPR: Major pathological response
CR: Complete response
PR: Partial response
SD: Stable disease
PD: Progressive disease
ORR: Objective response rate
DCR: Disease control rate
TRG: Tumor regression grade
PLR: Platelet-lymphocyte ratio
PNI: Onodera’s prognostic nutritional index
ICI: Immune checkpoint inhibitor
OR: Odd ratio
TRAEs: Treatment-related adverse events
POCs: Postoperative complications
HR: Hazard ratio
OS: Overall survival
RFS: Recurrence-free survival
ESPEN: European society for clinical nutrition and metabolism
ALB: Albumin
