# Supplementary figures and images for: Safety and efficacy of minimally invasive gastrectomy for older patients with gastric cancer after neoadjuvant chemotherapy and immunotherapy: a propensity score-matched analysis
Source: BMC Geriatr. 2024 Jul 15;24:606. doi: 10.1186/s12877-024-05193-w (PMC11251346; doi:10.1186/s12877-024-05193-w)

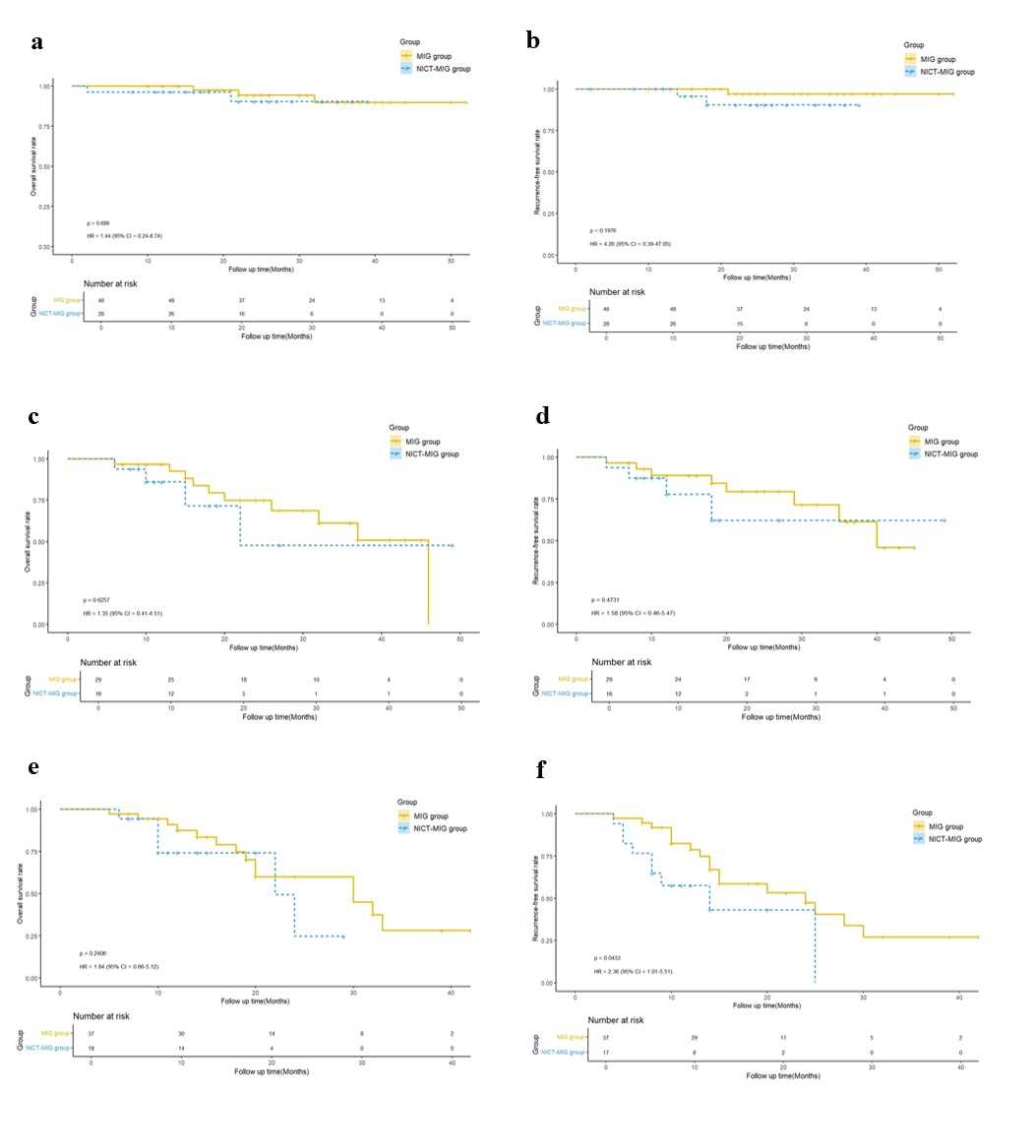

Supplement: Supplementary file 1 — Supplementary Material 1 [file 12877_2024_5193_MOESM1_ESM.jpg]

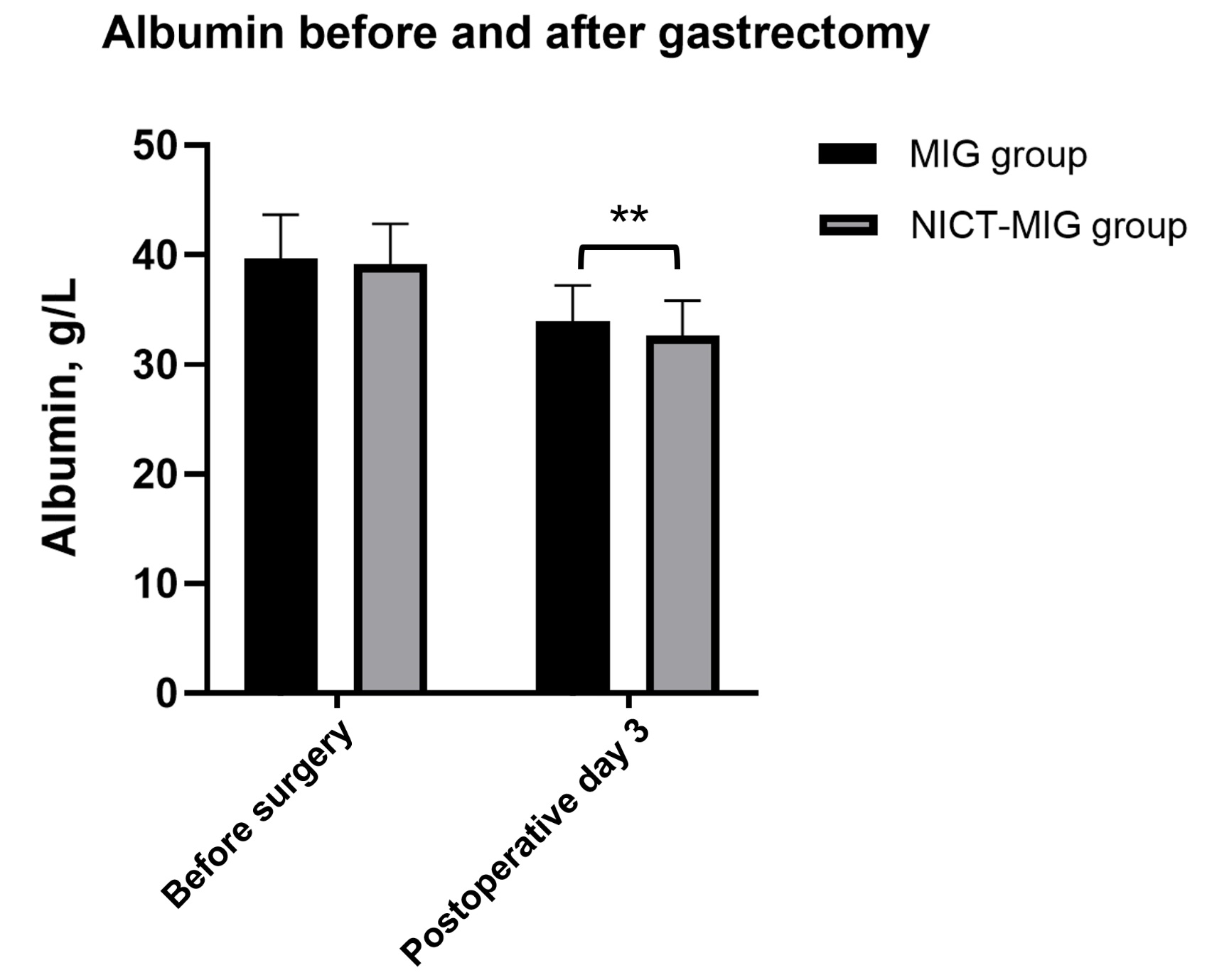

Supplement: Supplementary file 2 — Supplementary Material 2 [file 12877_2024_5193_MOESM2_ESM.jpg]
