# Supplementary material for: Safety and efficacy of minimally invasive gastrectomy for older patients with gastric cancer after neoadjuvant chemotherapy and immunotherapy: a propensity score-matched analysis
Source: BMC Geriatr. 2024 Jul 15;24:606. doi: 10.1186/s12877-024-05193-w (PMC11251346; doi:10.1186/s12877-024-05193-w)
Supplement: Supplementary file 3 — Supplementary Material 3 [file 12877_2024_5193_MOESM3_ESM.docx]

**Supplementary Table 1. Cox analysis for overall survival after MIG in older patients**

| **Factor** | **Univariate analysis** | | **P value** | **Multivariate analysis** | | **P value** |
| --- | --- | --- | --- | --- | --- | --- |
|  | **HR** | **95%CI** |  | **HR** | **95%CI** |  |
| Sex |  |  | 0.722 |  |  |  |
| Male | 1.000 |  |  |  |  |  |
| Female | 0.868 | 0.398-1.895 |  |  |  |  |
| Age (years) |  |  | 0.571 |  |  |  |
| ＜70 | 1.000 |  |  |  |  |  |
| ≥70 | 0.830 | 0.434-1.584 |  |  |  |  |
| NICT |  |  | 0.674 |  |  |  |
| No | 1.000 |  |  |  |  |  |
| Yes | 1.165 | 0.573-2.369 |  |  |  |  |
| NRS-2002 score |  |  | 0.879 |  |  |  |
| ＜3 | 1.000 |  |  |  |  |  |
| ≥3 | 0.948 | 0.478-1.880 |  |  |  |  |
| Surgical approach |  |  | 0.427 |  |  |  |
| Laparoscopic | 1.000 |  |  |  |  |  |
| Robotic | 1.466 | 0.570-3.775 |  |  |  |  |
| yp/pTNM stage |  |  | ＜0.001 |  |  | 0.001 |
| 0-I | 1.000 |  |  | 1.000 |  |  |
| II | 6.492 | 2.336-18.046 | ＜0.001 | 6.047 | 2.074-17.631 | 0.001 |
| III | 11.050 | 4.073-29.980 | ＜0.001 | 8.709 | 2.905-26.109 | ＜0.001 |
| R0 resection |  |  | 0.009 |  |  | 0.253 |
| No | 1.000 |  |  | 1.000 |  |  |
| Yes | 6.799 | 1.609-28.737 |  | 2.670 | 0.496-14.379 |  |
| Tumor resection |  |  | 0.463 |  |  |  |
| Proximal | 1.000 |  |  |  |  |  |
| Distal | 0.838 | 0.331-2.122 | 0.709 |  |  |  |
| Total | 1.345 | 0.610-2.965 | 0.463 |  |  |  |
| Tumor diameter(cm) |  |  | 0.002 |  |  | 0.321 |
| ＜3 | 1.000 |  |  | 1.000 |  |  |
| ≥3 | 3.263 | 1.541-6.908 |  | 1.567 | 0.645-3.804 |  |
| Albumin level(g/L) |  |  | 0.140 |  |  |  |
| ＜35 | 1.000 |  |  |  |  |  |
| ≥35 | 0.569 | 0.269-1.204 |  |  |  |  |
| PNI score |  |  | 0.282 |  |  |  |
| ＞45 | 1.000 |  |  |  |  |  |
| ≤45 | 1.431 | 0.745-2.747 |  |  |  |  |
| LMR |  |  | 0.022 |  |  | 0.015 |
| ＞3.25 | 1.000 |  |  | 1.000 |  |  |
| ≤3.25 | 2.154 | 1.115-4.163 |  | 2.358 | 1.184-4.694 |  |
| PLR |  |  | 0.495 |  |  |  |
| ＜148 | 1.000 |  |  |  |  |  |
| ≥148 | 1.255 | 0.654-2.405 |  |  |  |  |

**Abbreviation:** NICT: Neoadjuvant immunotherapy plus chemotherapy; MIG: Minimally invasive gastrectomy; BMI: Body mass index; aCCI: Age-adjusted Charlson Comorbidity Index; NRS-2002: Nutritional risk screening-2002; PLR: Platelet-lymphocyte ratio; LMR: Lymphocyte-monocyte ratio; PNI: Onodera’s prognostic nutritional index; HR: Hazard ratio

**Supplementary Table 2. Cox analysis for recurrence-free survival after MIG in older patients**

| **Factor** | **Univariate analysis** | | **P value** | **Multivariate analysis** | | **P value** |
| --- | --- | --- | --- | --- | --- | --- |
|  | **HR** | **95%CI** |  | **HR** | **95%CI** |  |
| Sex |  |  | 0.385 |  |  |  |
| Male | 1.000 |  |  |  |  |  |
| Female | 0.697 | 0.309-1.573 |  |  |  |  |
| Age (years) |  |  | 0.692 |  |  |  |
| ＜70 | 1.000 |  |  |  |  |  |
| ≥70 | 0.882 | 0.476-1.636 |  |  |  |  |
| NICT |  |  | 0.193 |  |  |  |
| No | 1.000 |  |  |  |  |  |
| Yes | 1.534 | 0.805-2.922 |  |  |  |  |
| NRS-2002 score |  |  | 0.846 |  |  |  |
| ＜3 | 1.000 |  |  |  |  |  |
| ≥3 | 1.069 | 0.545-2.096 |  |  |  |  |
| Surgical approach |  |  | 0.603 |  |  |  |
| Laparoscopic | 1.000 |  |  |  |  |  |
| Robotic | 1.282 | 0.502-3.276 |  |  |  |  |
| yp/pTNM stage |  |  | ＜0.001 |  |  | ＜0.001 |
| 0-I | 1.000 |  |  | 1.000 |  |  |
| II | 9.309 | 2.624-33.027 | 0.001 | 8.554 | 2.205-33.186 | 0.002 |
| III | 24.523 | 7.347-81.853 | ＜0.001 | 21.554 | 5.483-84.729 | ＜0.001 |
| R0 resection |  |  | ＜0.001 |  |  | 0.004 |
| No | 1.000 |  |  | 1.000 |  |  |
| Yes | 19.852 | 4.275-92.186 |  | 10.633 | 2.125-53.210 |  |
| Tumor resection |  |  | 0.023 |  |  | 0.831 |
| Proximal | 1.000 |  |  | 1.000 |  |  |
| Distal | 0.553 | 0.197-1.555 | 0.261 | 1.177 | 0.398-3.480 | 0.769 |
| Total | 1.786 | 0.835-3.819 | 0.135 | 1.283 | 0.574-2.865 | 0.544 |
| Tumor diameter(cm) |  |  | ＜0.001 |  |  | 0.874 |
| ＜3 | 1.000 |  |  | 1.000 |  |  |
| ≥3 | 4.010 | 1.848-8.704 |  | 1.073 | 0.452-2.546 |  |
| Albumin level(g/L) |  |  | 0.186 |  |  |  |
| ＜35 | 1.000 |  |  |  |  |  |
| ≥35 | 0.508 | 0.234-1.100 |  |  |  |  |
| PNI score |  |  | 0.220 |  |  |  |
| ＞45 | 1.000 |  |  |  |  |  |
| ≤45 | 1.476 | 0.792-2.750 |  |  |  |  |
| LMR |  |  | 0.224 |  |  |  |
| ＞3.25 | 1.000 |  |  |  |  |  |
| ≤3.25 | 1.487 | 0.785-2.819 |  |  |  |  |
| PLR |  |  | 0.457 |  |  |  |
| ＜148 | 1.000 |  |  |  |  |  |
| ≥148 | 1.269 | 0.677-2.378 |  |  |  |  |

**Abbreviation:** NICT: Neoadjuvant immunotherapy plus chemotherapy; MIG: Minimally invasive gastrectomy; BMI: Body mass index; aCCI: Age-adjusted Charlson Comorbidity Index; NRS-2002: Nutritional risk screening-2002; PLR: Platelet-lymphocyte ratio; LMR: Lymphocyte-monocyte ratio; PNI: Onodera’s prognostic nutritional index; HR: Hazard ratio
